# The influence of environmental factors on the job burnout of physical education teachers in tertiary education

**DOI:** 10.1038/s41598-024-59748-3

**Published:** 2024-04-21

**Authors:** KunZhan Li, XiaoShu Xu, YunFeng Zhang, XinYu Xu

**Affiliations:** 1 School of Physical Education, Zhengzhou Technology and Business University, Zhengzhou, China; 2 School of Foreign Studies, Wenzhou University, Wenzhou City, Zhejiang Province China; 3 Faculty of Languages and Translation, Macao Polytechnic University, R. de Luís Gonzaga Gomes, Macao, China

**Keywords:** Environmental factors, Job burnout, Physical education, Tertiary education, Psychology, Engineering

## Abstract

This study takes environmental factors and individual factors as variables to explore the deep internal mechanism of the impact of a comprehensive environment on higher education physical education (PE) teachers’ job burnout. Little research has been done on how environmental factors affect the internal mechanism of college and university PE teachers’ job burnout through individual factors (e.g., professional pressure and teaching efficacy). In this study, the participants were 231 PE teachers from seven comprehensive universities, and four questionnaires were administered to measure the participants’ job burnout, perceived overall environment, teaching efficacy, and occupational stress. Research has found that environmental factors have a significant negative impact on occupational stress, and occupational stress plays an important mediating role between environment and occupational burnout. Research has shown that differences in external environments lead to varying levels of personal stress among college physical education teachers, which in turn affects their level of occupational burnout. The study concludes that a good social, working, and living environment helps to reduce the work pressure on PE teachers, improves their sense of teaching efficacy, and inhibits the occurrence of teachers’ job burnout.

## Introduction

Job burnout refers to the physical pressure and mental effort of employees to adapt to a demanding working environment^1^. Teachers’ job burnout has been described by Kyriacou as a syndrome caused by long-term teacher stress^2^, which is characterized by emotional, physical, and attitudinal fatigue. Poor occupational structure, low salary, and poor working conditions have been reported as the main factors leading to teachers’ dissatisfaction with their jobs and their willingness to leave^3^. The level of self-efficacy, social support, and job demand have significant effects on teachers’ job burnout^4^. First of all, self-efficacy and burnout have a positive direct impact: the higher the teacher’s self-efficacy, the higher the sense of burnout^5^. Because high self-efficacy can enable teachers to master multiple aspects of work at the same time, they feel tired. Secondly, teachers’ job burnout is positively related to job demand: the higher the job demand, the higher the burnout; and the lower the job demand, the lower the degree of job burnout^6^. Third, higher social support can lead to a higher sense of burnout. This is because while friends, peers, colleagues, leaders, and family are there to encourage or strongly support them^7^, the fact that these other people hope or expect teachers to complete their work on time and according to expectations and support goals will make them feel overwhelmed when performing and completing tasks.

Job burnout will not only affect the development of individual teachers but will also have a negative impact on higher institutions, students, and society. In addition to individual factors, environmental factors also have a significant impact on teachers’ job burnout^8^. For teachers, the professional environment mainly includes higher institutions’ environment, social environment, and family environment. Support from higher institutions and society can effectively alleviate teachers’ job burnout^9^. Previous research on the relationship between teachers’ professional environment and job burnout focuses mainly on the school environment^10^, however, school management, teacher-student relationships and other factors are closely related to teachers’ job burnout. For example, college teachers who have good interpersonal relationships have comparatively lower job burnout. Meanwhile, when teachers face pressure from numerous aspects, such as workload, examination pressure, and student management, it is likely to result in a sense of powerlessness and even frustration due to the limited resources. Previous studies have indicated that there are positive relationships between the occupational stress and teacher burnout. Previous studies have also indicated that there are positive relationships between the occupational stress and teacher burnout^11^ Other environmental factors such as lack of social support and poor interpersonal relationships can also be sources of teachers’ stress^12^, and the greater the pressure, the more serious the job burnout of college teachers. The school environment, social environment, and the support of family and friends have a certain positive predictive effect on the teaching efficacy of college PE teachers, while self-efficacy is significantly negatively correlated with job burnout^10^. Job stress not only directly leads to job burnout, but also indirectly affects job burnout through teaching efficacy; that is, teaching efficacy plays an intermediary role between job stress and job burnout^13^.

Occupational stress refers to the unpleasant negative emotional experience experienced by teachers, which may lead to excessive physical and mental fatigue, nervousness, depression, or pain due to factors such as long working hours, heavy workload, and serious inappropriate student behavior^14^. Research shows that teaching is one of the most stressful professions. With the reform of education and the passage of time, the sources of work pressure for primary and secondary school teachers are also changing^15^. Firstly, teachers need to change their previous teaching content and work methods in accordance with national policy requirements, which undoubtedly increases their workload. Secondly, teachers face pressure from schools to evaluate their teaching quality and performance. On the other hand, burnout is conceptualized as “a state of physical, emotional, and mental exhaustion caused by long-term involvement in emotionally demanding work environments”. It has been unanimously proven that chronic stress is implicit in the development of fatigue. Stress itself is believed to develop from the interaction between environmental and personal factors^16^.

Although there have been many studies on job satisfaction over the past few decades, there is little research on the job burnout of physical education teachers and how environmental factors affect it. There is a wealth of research on the influencing factors of teacher burnout. In addition to individual factors, environmental factors also have an important impact on teacher burnout. The professional environment of teachers mainly includes school environment, social environment, and family environment. School and social support can effectively alleviate the professional burnout of teachers. Most research on the relationship between teacher professional environment and occupational burnout focuses on the school environment, and factors such as school management and teacher-student relationships are closely related to teacher occupational burnout^17^. When exploring the relationship between environmental factors and job burnout, there is also little research on mediating influencing factors. It has been reported that the factors most consistently associated with teacher burnout in our study were teachers’ perceptions of the school’s safety and support and student attitudes to learning^18^. In terms of sociodemographic variables, gender and rural/urban teaching environment did not have significant impact on teacher burnout profiles, but professional experience did^19^. A recent reported has indicated that PE teachers have different levels of burnout, and their physical, organizational, and socio-cultural resources are closely related to their level of burnout. The needs that cause fatigue and stress have been identified as paperwork and bureaucracy, student related factors, and experiences related to the pandemic^20^. Therefore, this study aims to start from environmental factors, using individual factors as indirect influencing variables, and explore the deep-seated internal mechanism of the impact of comprehensive environment on the occupational burnout of college physical education teachers. More specifically, the first attempt is to explore the indirect effects of occupational stress and teaching efficacy in environmental factors on the occupational burnout of university physical education teachers. On this basis, the comprehensive effects of occupational stress and teaching effectiveness on the occupational burnout of college physical education teachers were further explored. Finally, strategies were proposed to reduce or avoid occupational burnout among physical education teachers. Three hypotheses are put forward as follows:

### H1

Occupational stress plays an intermediary role in the influence of environmental factors on the job burnout of college PE teachers.

### H2

Teaching efficacy plays a mediating role in the influence of environmental factors on college PE teachers’ job burnout.

### H3

Occupational stress and teaching efficacy play a chain intermediary role in the influence of environmental factors on college PE teachers’ job burnout.

## Methods

### Participants

The research design used a cross-sectional survey. In this study, a stratified sampling design was used to collect data from participants. Stratified sampling is considered the best method for collecting data because of its simple concept. Sampling is based on the assumption that each item in a group has an equal chance of being selected in the sample. Layered sampling is considered a reasonable method for collecting data in this study, as it ensures the accuracy of parameter estimation and the representativeness of each sample. Therefore, based on the 2020 China University Soft Science Ranking (Shanghai Ranking), we uniformly divide universities in Henan Province into five levels, which use hundreds of indicator variables (including research output and reputation factors) to comprehensively evaluate Chinese universities. The participating colleges and universities are randomly selected from each class. The guidelines for randomly selecting schools from each class are: (a) all classes are clearly distinguished from other classes, (b) all data for each class is consistent, which means that the samples selected from each class will represent the schools of that class. In order to evaluate the representativeness of the sample in demographic data, the overall demographic data (2019) was obtained from the Ministry of Education of China. Snowball sampling and criterion sampling methods were employed in the study.

The first author of this study received ethics approval from the Ethics Committee for Human Research, Zhengzhou Technology and Business University, and all methods were carried out in accordance with relevant guidelines and regulations. The participants in this study were informed of the research objectives two weeks before the interview. They were invited to provide data through an online questionnaire. Informed consent was obtained through the web-based survey software “Manjushin” provided by sojump.com. All survey responses were anonymous and all participation in the study was entirely voluntary. Participants were informed that completion of the survey would not have a direct impact on them, either in terms of gain or loss, as all data collected would be aggregated and disclosed in aggregate form only. When participants clicked on a survey, they were prompted to a page containing brief information about the survey and their rights to privacy and anonymity. They then just needed to click “Agree to Continue” to indicate their consent. The link shared with participants includes a questionnaire and instructions for filling it out. It also includes guidelines that inform teachers that their participation will be voluntary, anonymous, and confidential. Participants can fill out questionnaires on the online platform as needed. The inclusion criteria for participants were as follows: (1) currently working full-time at the selected institutions; and (2) have participated in PE teaching within the previous year. The criteria for elimination include: (a) regular reaction patterns; (b) Missing data; (c) Contradictory reactions to related projects (such as inconsistent reactions to homogeneous projects or consistent reactions to opposing projects). Benter and Chou indicated that the sample size should be 10 times the number of variables in the analysis^21^. Thus, the minimum sample size in our study was 200 which was considered sufficient to provide good statistical power.

Altogether, 240 college PE teachers from seven colleges and universities in Henan Province, China, took part in this study through the “Wenjuan Xing” platform on March 10–May 10, 2023. Among the 240 returned questionnaires, 231 were valid and 9 questionnaires were rejected. Among the valid responses, male teachers accounted for 64% and female teachers 36%; 77% of teachers were equipped with a graduate degree or above, and 23% an undergraduate degree; 39% of the participants had ten years’ working experience, 30% had worked for three to ten years, 22% had more than 21 years’ working experience, and the remaining 9% had less than two years’ working experience; and in terms of teachers’ professional position, professors accounted for 4%, associate professors for 19%, lecturers for 67%, and teaching assistants for 10%.

### Measures

To have a comprehensive understanding of the current situation of teachers’ job burnout, and to explore the causes and influencing mechanism of teachers’ job burnout from environmental factors and individual factors, this study used four questionnaires to measure the degree of job burnout, the perceived overall environment, the level of teaching efficacy and the degree of professional pressure on college PE teachers. All questionnaires, originally in English, were translated into Chinese by a bilingual translation expert. They were then pilot tested for clarity and understandability with ten undergraduates, ensuring their validity and reliability.

*Teachers’ job burnout* adopted the Teacher Job Burnout Questionnaire (MBI-ES) compiled by Maleshi et al., which included three dimensions: emotional exhaustion (EE), depersonalization (DP), and low personal achievement (PA), with 21 questions. A 5-point scoring method was adopted, including: very non-conforming (1 point), relatively non-conforming (2 points), generally conforming (3 points), relatively conforming (4 points), and very conforming (5 points). The higher the score, the higher the degree of burnout. The questionnaire is considered to have good reliability, validity, and cross-cultural consistency. The internal consistency reliability is 0.83.

*The environmental factors* adopted the questionnaire compiled by previous study, which included the support of school, society, family, and friends. The school environment was composed of the following five dimensions: the influence of the principal, the development conditions provided by work, the school atmosphere, interpersonal relationships, and the material environment. There were 18 questions in total, with a 5-point scoring method, including: completely unqualified (1 point), unqualified (2 points), basically qualified (3 points), relatively qualified (4 points), and very qualified (5 points). The higher the score, the better the school environment. The questionnaire is widely used and is considered to have good reliability and validity. The internal consistency reliability of this study is 0.80.

*Teachers’ teaching efficacy* adopted the scale (TES) prepared by Yu et al., which included two dimensions: personal teaching efficacy (17 questions) and general education efficacy (10 questions). There were 21 questions, with a 4-point scoring method, including: completely correct (1 point), mostly correct (2 points), somewhat incorrect (3 points), and completely incorrect (4 points). The higher the score, the higher the teacher’s teaching efficacy. The internal consistency coefficient of the total table is 0.77, and the internal consistency coefficient of this study is 0.72.

*College teachers’ occupational stress* adopted the scale prepared by Li. The questionnaire included five dimensions: job security, teaching security, interpersonal relationship, workload, and work fun, with a total of 24 questions. It adopted a 4-point scoring method, including no pressure (1 point), mild pressure (2 points), moderate pressure (3 points), and severe pressure (4 points). The higher the score, the greater the pressure. The questionnaire is considered to have good reliability, and the internal consistency reliability of this study is 0.87.

### Procedure

**Figure 1:**
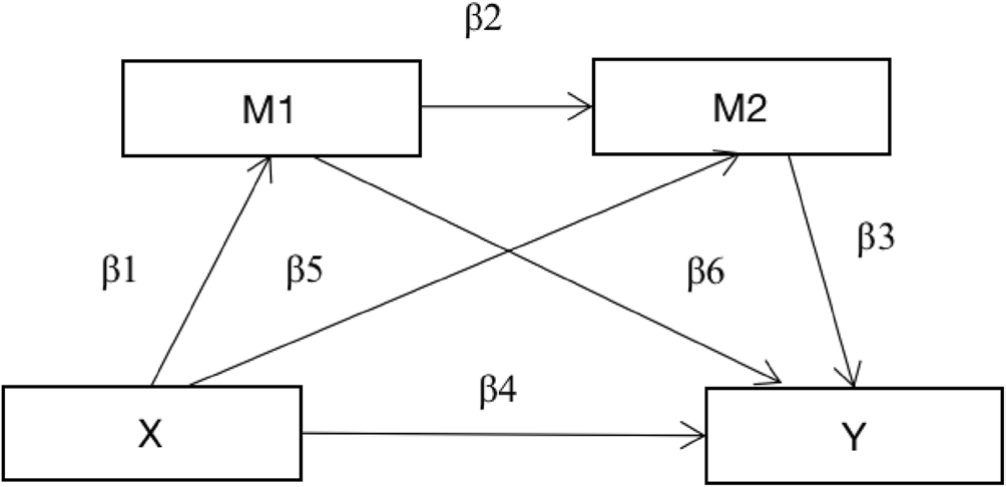
Chain mediation model.

Chain intermediary model was employed for this study. The collected data were processed in Zhengzhou, Henan Province, China. SPSS 22.0 software was used for descriptive statistical analysis, regression analysis and chain-mediated effect analysis. Normality of collected data was tested using the Kolmogorov–Smirnov test. Firstly, descriptive statistics and correlation analysis were conducted in SPSS 21.0 to explore the correlation between key variables. Secondly, the assumed model was tested using structural equation modeling (SEM) in Mplus 8.0. The data analysis for this study was divided into four steps: first, the common method deviation of the data was tested; second, the job burnout and the environment of college PE teachers were described and counted; third, regression analysis was used to explore the predictive relationship between environmental factors, occupational stress, teaching efficacy and job burnout; and fourth, the chain intermediary model between occupational stress and teaching efficacy was tested in the relationship between environmental factors and teachers’ job burnout. Chain mediation refers to how multiple intermediary variables show sequential characteristics, and the predictive variables have indirect effects on the outcome variables through the intermediary chain. Chain mediation can better reveal the complex internal mechanism of the relationship between variables. In the chain model below, X represents the predictive variable (environmental factors), M1 represents the intermediary variable 1 (occupational stress), M2 represents the intermediary variable 2 (teaching efficacy), and Y represents the dependent variable (job burnout). Based on the results of the national education survey, a confirmatory factor analysis (CFA) with maximum likelihood estimation was conducted to examine potential factor structures^22^. CFA utilized the Satora Bentler (SB) robust scaling method. The model fitting used the following four different methods: (1) SB scale chi square (χ^2^ SB); (2) Standardized root mean square residuals; (3) The approximate root mean square error (RMSEA) of the SB scale; comparison fit index (CFISSB) with (4) SB Scale. These indices are used to determine whether the exported model conforms to the data. The following criteria are used to evaluate model fit: SRMR ≤ 0.08, RMSEA ≤ 0.08, CFI ≥ 0.90. After CFA, evaluate these factors to ensure they account for sufficient variance in the response. The reliability of the initial queues for EFA and CFA was evaluated using Cronbach α. The reliability of inventory generated by CFA is calculated as a coefficient ω.CFA was conducted in R version 4.1.1 using lavaan latent variable analysis package version 0.6.8^23^. The model includes three mediation paths: (1) β1β6, (2) β5β3, and (3) β1β2 β3 (Fig. 1).

## Results

This study used self-report to collect data, which may have common methodological bias. Harman single factor test method was used to test the common method deviation of all variables. The results showed that the characteristic root of 22 factors was greater than 1, the first factor could only explain the critical standard of 20.79%, and less than 40% and the common method deviation was not significant.

From the average value of each variable, the average value of the three dimensions of job burnout was between 1.88 and 2.66; the average value was less than 3 points, indicating that the degree of job burnout was low. From the mean value of the three dimensions, the score of emotional exhaustion was the highest and the score of de-personalization was the lowest, indicating that college PE teachers had a certain degree of emotional exhaustion and lack of personal achievement. This result reflected the excessive emotional effort of college PE teachers and showed a lack of self-identity. Among the environmental factors, the family environment had the highest score, followed by the social environment, and the school environment had the lowest score. The score for the principal’s influence in the school environment was the lowest, which indicates that college leaders did not care enough about the work of PE teachers, resulting in the emergence of a low sense of personal achievement. The scores of each dimension of occupational stress were between 2.01 and 2.5, indicating that the overall level of occupational stress of PE teachers was not too serious. Among all dimensions, the score of the workload dimension was the largest and the score of the interpersonal relationship dimension was the lowest, indicating that the workload of college PE teachers was the main source of stress. The average value of PE teachers’ personal teaching efficacy was 4.35, and the average value of general education efficacy was 3.82, indicating that the overall level of PE teachers’ personal teaching efficacy was good. From the correlation between variables, job burnout had a significant negative correlation with environmental factors and teaching efficacy, and a significant positive correlation with occupational stress. This showed that environmental factors, occupational stress and teaching efficacy had an significant impact on teachers’ job burnout (Table 1).

**Table 1:** Descriptive statistics and correlation analysis of each research variable.

M	M	SD	1	2	3	4	5	6	7	8
1. School environment	3.08	0.50	1							
2. Social environment	3.31	0.81	0.52***	1						
3. Family environment	3.57	0.84	0.37***	0.49***	1					
4. Occupational stress	11.36	2.58	− 0.35***	− 0.27***	− 0.34***	1				
5. Teaching effectiveness	8.19	1.31	0.28***	0.16***	0.10	− 0.28***	1			
6. Emotional exhaustion	2.66	0.55	− 0.37***	− 0.11***	− 0.17*	0.61***	− 0.41***	1		
7. Depersonalization	1.88	0.56	− 0.25***	− 0.07***	− 0.13	0.41***	− 0.45***	0.63***	1	
8. Low sense of achievement	2.33	0.51	− 0.23***	− 0.12	− 0.26***	0.39***	− 0.57***	0.55***	0.61***	1

*p < 0.005, **p < 0.01, ***p < 0.001.

### Regression analysis of variables

The correlation between the variables was significant, indicating that the correlation was meaningful. Regression analysis can be used to further explore the relationship between variables; this study took job burnout as the dependent variable and environmental factors, occupational stress, and teaching efficacy as the independent variables for regression analysis. Relationship between Occupational Stress and Environmental Factor was caculated as the following: Occupational Stress = β_1_ * Environmental Factor + ε, where β_1_ = − 0.31 (Standard Error = 0.05, t = − 6.63, *p* < 0.001). Relationship between teaching Effectiveness and both Environmental Factor and Occupational Stress was caculated as the following: Teaching Effectiveness = β_2_ * Environmental Factor + β_3_ * Occupational Stress + ε, where β_2_ = 0.08 (Standard Error = 0.03, t = 2.72, *p* < 0.01) β_3_ = − 0.09 (Standard Error = 0.04, t = − 2.51, *p* < 0.005). Relationship between Job Burnout and Environmental Factor, Occupational Stress, and Teaching Effectiveness was caculated as the following: Job Burnout = β_4_ * Environmental Factor + β_5_ * Occupational Stress + β_6_ * Teaching Effectiveness + ε, where β_4_ = − 0.02 (Standard Error = 0.02, t = − 0.67, *p* > 0.05) β_5_ = 0.22 (Standard Error = 0.03, t = 7.28, *p* < 0.001) β_6_ = − 0.45 (Standard Error = 0.06, t = − 7.97, *p* < 0.001). The analysis found that the direct predictive effect of environment on job burnout was not significant (β = − 0.02,* p* > 0.05), but it could negatively predict occupational stress (β = − 0.31, *p* < 0.001), and it could positively predict the sense of teaching efficacy (β = 0.08, *p* < 0.001). In addition, occupational stress could positively predict job burnout (β = 0.22, *p* < 0.001), and negatively predict teaching efficacy (β = − 0.09, *p* < 0.001). Teaching efficacy negatively predicted job burnout (β = − 0.45, *p* < 0.001) (Table 2).

**Table 2:** Regression Analysis of the relationship between variables in the model.

Regression equation		Overall fitting index			Significance of regression coefficient		
Result variable	Predictive variables	R	R^2^	F	β	SE	t
Occupational stress	Environmental factor	0.43	0.19	23.57	− 0.31	0.05	− 6.63***
Teaching effectiveness	Environmental factor	0.34	0.11	8.57	0.08	0.03	2.72**
	Occupational stress				− 0.09	0.04	− 2.51*
Job burnout	Environmental factor	0.69	0.48	46.33	− 0.02	0.02	− 0.67
	Occupational stress				0.22	0.03	7.28***
	Teaching effectiveness				− 0.45	0.06	− 7.97***

*p < 0.005, **p < 0.01, ***p < 0.001.

### The relationship between environmental factors and job burnout

**Figure 2:**
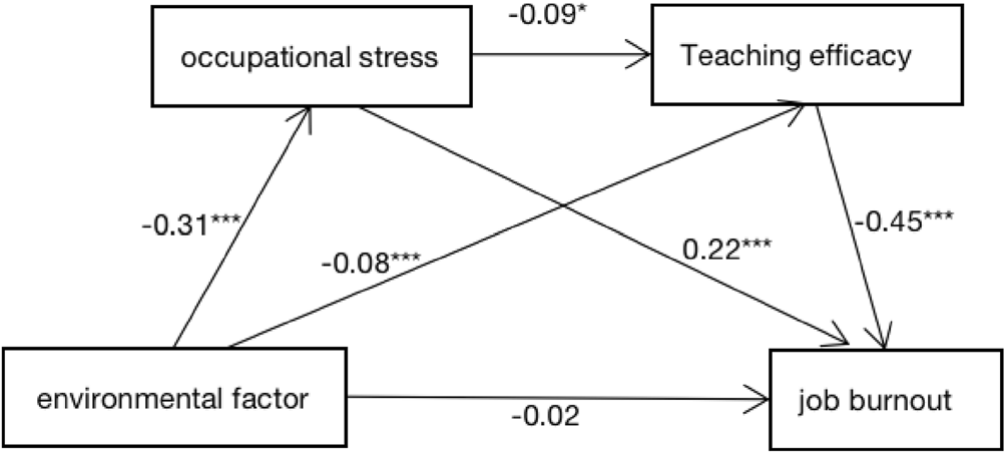
The chain mediating effect of occupational stress and teaching efficacy.

In order to further investigate the relationship between environmental factors and job burnout of college PE teachers, this study used SPSS macro compiled by Hayes to analyze the intermediary role of occupational stress and teaching efficacy in the impact of environmental factors on job burnout (Fig. 2).

Occupational stress and teaching efficacy played an intermediary role between environment and job burnout. The mediating effect was composed of three indirect effects: indirect effect 1 (− 0.070) through environment → occupational stress → job burnout; indirect effect 2 (− 0.034) by way of environment → teaching efficacy → job burnout; indirect effect 3 (− 0.013) produced by way of environment → occupational stress → teaching efficacy → job burnout. The three indirect effects accounted for 52.58%, 25.77%, and 10.04% of the total effects, respectively. The bootstrap 95% confidence interval of the three paths did not contain 0, indicating that the three indirect effects reached a significant level. The results showed that the chain mediation effect of occupational stress and teaching efficacy was significant, and the mediation effect of the three paths was different (Table 3).

**Table 3:** An analysis of the mediating effect between occupational stress and teaching efficacy.

	Indirect effect value	Boot standard error	Boot CI lower limit	Boot CI lower limit	Relative mediating effect (%)
Total indirect effect	− 0.118	0.023	− 0.166	− 0.076	88.31
Indirect effect 1	− 0.070	0.015	− 0.102	− 0.044	52.58
Indirect effect 2	− 0.034	0.016	− 0.067	− 0.006	25.77
Indirect effect 3	− 0.013	0.006	− 0.026	− 0.002	10.04

Boot standard error, Boot CI lower limit and Boot CI upper limit refer to the standard error of indirect effects estimated by the deviation corrected percentile Bootstrap method, and the lower and upper limits of 95% confidence intervals, respectively.

## Discussion

This study aims to use quantitative methods, including four questionnaires, to investigate the impact of comprehensive environment on job burnout of college physical education teachers, in order to measure the degree of job burnout, overall environmental perception, teaching effectiveness level, and occupational stress level of college physical education teachers. Research has found that environmental factors have a significant negative impact on occupational stress, and occupational stress plays an important mediating role between environment and occupational burnout. Research has shown that differences in external environments lead to varying levels of personal stress among college physical education teachers, which in turn affects their level of occupational burnout. Environmental factors often considered as sources of teacher stress include external demands such as excessive workload, time pressure, lack of resources, paperwork, student behavior Organizational factors such as leadership support level and school atmosphere, as well as reviews around teacher efficacy^24^. Inconsistent results were reported when studying stress in the teaching population. For example, several studies have shown that early career teachers face a high risk of stress and burnout, and this risk is increasing. However, other researchers believe that the risk of burnout does not vary based on experience^25^. At various school levels, some studies have shown that primary school teachers experience greater stress and burnout than high school teachers, while OECD research shows that the trend is opposite across countries, indicating no significant overall difference in education levels^26^. Rajendran et al. found that there was no difference in the levels of emotional exhaustion and burnout between primary and secondary school teachers^27^, while De Nobile and McCormick did not distinguish between primary and secondary school teachers, but found that classroom teachers had greater stress than any other type of educator. Finally, some preliminary evidence suggests that teachers in urban areas report greater stress compared to rural teachers, but there is no difference in the level of burnout among these groups^28^. These evidences indicate that the environmental factors that affect the teaching pressure of college physical education teachers are the changes in their social environment and actual social status in terms of income and life, economic income status, family harmony, as well as the changes in the material and cultural environment of the school environment. However, when the pressure brought by the environment exceeds the capacity of teachers, if not effectively eliminated in a timely manner, it can lead to work fatigue. Therefore, this study indicates that a good social, school, and family environment is conducive to college physical education teachers actively facing work and life problems, thereby alleviating occupational stress and reducing the possibility of occupational burnout.

Teaching efficacy has a negative predictive effect on job burnout, and also plays a mediating role between environmental factors and job burnout. Previous studies have shown that the lower the sense of teaching effectiveness, the more severe the degree of job burnout^28^. A study targeting high school teachers found that one-third of them experience a high level of fatigue. The study also found evidence that an increase in fatigue is associated with low perceived efficacy (individual and collective) scores, low job satisfaction, and low career commitment. Furthermore, when teachers experience high levels of fatigue, their perception of the educational environment becomes less positive. Finally, the research findings indicate that perceived personal efficacy mediates the relationship between burnout and job satisfaction^29^. The results of this study further indicate that the environment can lead to an improvement or decrease in teaching effectiveness, and the improvement of teaching effectiveness has a positive impact on alleviating work fatigue. Therefore, when college physical education teachers feel that society and schools value sports and respect their profession, these teachers can recognize the importance of their profession, which will enhance their confidence and enable them to actively and effectively solve teaching problems, thereby reducing work fatigue. On the contrary, university physical education teachers who believe that physical education courses are marginalized by society and schools will have lower confidence. College physical education teachers with low self-efficacy may have unrealistic understanding of their subjective judgment of abilities and may doubt whether they can teach students well or handle relationships with them well. As a result, they were unable to complete their satisfactory work, leading to low mood Occupational stress and teaching effectiveness play a series of multiple mediating roles in the relationship between environmental factors and occupational burnout; Environmental factors affect the occupational burnout of college physical education teachers through the combined effect of occupational stress and teaching effectiveness. This discovery effectively validates the stress cognitive interaction theory, which emphasizes that work stress is an interactive process between individuals and the environment. Through personal cognitive assessment, potential stressors can become actual stressors, mainly influenced by self-efficacy. This means that individuals with low self-efficacy will convert potential sources of stress into actual stress. Based on the theory of stress cognitive interaction, the results of this study indicate that positive environmental factors can alleviate teachers’ occupational stress, enhance their problem-solving ability, improve their self-efficacy, and thus avoid professional burnout.

The survey results indicate a significant correlation between environmental factors, occupational stress, teaching effectiveness, and job burnout. The regression analysis results of this study further indicate that environmental factors do not have a significant direct predictive effect on job burnout, but have a significant predictive effect on occupational stress and teaching effectiveness. Both occupational stress and educational effectiveness can affect job burnout, further indicating a close relationship between variables. It has been found that teachers’ physical, organizational, and socio-cultural resources are closely related to their level of burnout. The needs that cause fatigue and stress have been identified as paperwork and bureaucracy, student related factors, and experiences related to the pandemic^30^. These findings indicated the complexity of involved factors contributing to burnout. Therefore, the intermediary effect analysis was used to further explore the relationship between variables, and it is found that the intermediary effect is produced through three indirect ways: through the independent effect of occupational stress; through the independent role of teaching efficacy; and through the joint effect of professional pressure and teaching efficacy.

## Conclusion

### Summary of main findings

This research delves into the profound psychological condition of burnout, a phenomenon with far-reaching implications for individuals and institutions, particularly within educational contexts such as schools. Drawing upon the framework of event system theory, this study thoroughly investigates the profound influence of environmental factors on job burnout among physical education (PE) teachers in colleges and universities. Additionally, it delves into the intricate psychological mechanisms that underpin this relationship.

To answer the research question “How do environmental factors, occupational stress, and teaching effectiveness interact to shape job burnout among physical education (PE) teachers in higher education?” the study enlisted 231 PE teachers from seven comprehensive universities as participants. They provided valuable insights through the completion of four questionnaires measuring job burnout, perceived overall environment, teaching efficacy, and occupational stress.

The survey outcomes unveiled compelling evidence of a substantial correlation among environmental factors, occupational stress, teaching effectiveness, and job burnout among PE teachers in higher education. These findings emphasize the multifaceted dynamics inherent in the academic environment.

Significantly, the regression analysis illuminated these relationships further. Particularly noteworthy is the significant influence of environmental factors on both occupational stress and teaching effectiveness. While environmental factors themselves may not directly foretell job burnout, their role in shaping the experiences of PE teachers within college and university settings is pivotal.

Furthermore, the study identified occupational stress and teaching effectiveness as critical determinants of job burnout, highlighting the intricate interdependence among these variables. This underscores the significance of environmental factors as indirect contributors to the multifaceted phenomenon of job burnout.

### Implications

The findings of this study have profound industrial implications, particularly in highlighting the intricate link between psychological distress and physical health outcomes. The established connection between chronic psychological stress and physiological conditions such as hypertension, obesity, and other negative health behaviors underlines the urgent need for workplace interventions. These conditions contribute significantly to the global burden of cardiovascular diseases and overall mortality rates. Specifically, the research draws attention to the dire consequences of burnout as evidenced by the Maslach Burnout Scale, where high levels of burnout and emotional exhaustion are shown to markedly increase the risk of mortality.

The implications extend beyond educational settings, touching on various sectors where occupational stress is prevalent, notably in healthcare. The necessity for additional research into stress management techniques, such as Transcendental Meditation (TM), is underscored for environments where stress is an inherent part of the job. This not only highlights the need for sector-wide changes but also calls for a reassessment of organizational health policies to encompass mental well-being strategies.

Moreover, the study reveals that the perception of work stressors significantly influences health outcomes. This brings to light the importance of psychological resilience and perception management in mitigating occupational stress. Consequently, there is a compelling case for industries to invest in developing interventions that enhance career engagement and reduce perceived threats from the work environment. Such interventions should not only aim to modify environmental stressors but also bolster individual coping mechanisms, thereby improving overall job satisfaction and mental health.

Therefore, it is imperative for organizations, especially those in high-stress industries, to adopt a holistic approach to employee well-being. This involves creating supportive environments that reduce occupational stress and promote teaching effectiveness, thereby fostering better mental and physical health among employees. In particular, for Physical Education teachers, developing targeted interventions to enhance their sense of vocational calling and to manage stress could significantly alleviate job burnout, leading to improved educational outcomes and healthier work-life balances. The broader industrial implication is clear: a healthier workforce leads to a more productive and sustainable organizational environment.

### Recommendations

To effectively mitigate educator burnout, it is imperative that organizations prioritize the enhancement of both the ecological and organizational environments within educational settings. This encompasses a concerted effort from both governmental bodies and educational institutions to integrate the experiences and insights of educators into the formulation of health and well-being policies. Emphasizing a bottom-up approach, where policies are informed by the firsthand experiences of those within the educational sector, could prove more efficacious than traditional top-down mandates. This approach should be complemented by capitalizing on the unique benefits and characteristics inherent to the sports discipline, encouraging peer exchanges, and fostering innovation and pedagogical reform across comparable institutions.

In addressing the issue of excessive educator workloads, a significant increase in the recruitment of qualified and experienced physical education teachers is essential. This strategy aims to distribute workloads more evenly, thereby preventing burnout resulting from overwork. Additionally, there is a critical need for the restructuring of evaluation and rewards systems within universities to better reflect the unique challenges faced by physical education teachers. Tailoring these systems to acknowledge the distinct nature of physical education will alleviate stress and contribute to a more rewarding career path.

Further, educational institutions must reconsider their criteria for professional advancement, especially under the unique demands of physical education. Ensuring adequate opportunities for career development can significantly reduce the anxiety associated with professional growth and job security. This should be coupled with proactive measures to manage and reduce work-induced stress, including the development of comprehensive strategies to identify and counteract the early signs of resource depletion and burnout among educators.

Finally, it is crucial to establish well-being and rehabilitation programs aimed at fostering psychological recovery and reducing teacher fatigue. Such initiatives should focus on enabling psychological detachment, promoting relaxation, and providing control mechanisms to manage work-related stress effectively. Supporting these programs with flexible work arrangements can offer educators the necessary space to recover from the demands of their profession, thereby enhancing overall job satisfaction and effectiveness.

By implementing these multi-faceted recommendations, educational institutions and policymakers can create a more supportive environment that not only improves the well-being and efficacy of physical education teachers but also positively impacts the educational experience for students.

### Limitations

This study, while offering positive insights and contributions to theory and practice, faces several limitations. Firstly, the COVID-19 pandemic restricted interaction with participants, leading to a small sample size that may not fully represent the broader population. Despite using stratified sampling based on university rankings, the sample size’s limitations suggest the need for future studies to collect more data or employ different sampling methods, like simple random sampling. Secondly, the sample exclusively comprising Chinese higher education lecturers limits the generalizability of findings to other groups with distinct organizational cultures and expectations, such as those in the global higher education sector. This highlights the potential impact of cultural differences on factors like burnout or turnover intention, suggesting future research should explore the role of cultural background in job satisfaction and proactive personality regulation. Additionally, the study’s cross-sectional design hinders causal inferences about the relationship between work stress and burnout, indicating a need for longitudinal studies to establish stronger causal evidence. Another limitation is the lack of differentiation in individual factors like gender and teaching experience. Addressing this gap is crucial for understanding how resilience buffers teachers against negative work-related issues and contributes to their sustainable professional development.

### Focus of future study

Further empirical research, especially qualitative research, can be conducted through intermediary tools to gain a better understanding of the role of educational worker burnout. Further experience and educational exams are crucial in investigating the mediating role of reducing burnout among educators. Further research should be conducted on the impact of career development and related educational factors to clarify their long-term impact on education.It is recommended that university leaders pay more attention to the important role of sports in the development of universities, take practical and feasible measures, and avoid the marginalization of sports in disciplinary construction, evaluation of key talents, and scientific research rewards.

## Data Availability

The data that support the findings of this study are available from the authors; however, restrictions apply to the availability of these data, so they are not publicly available. Interested researchers (who meet the criteria for access to confidential data) may contact the corresponding author of this paper for access to the datasets generated or analyzed during the current study.
